# Elderly suicide rates: a replication of cross-national comparisons and association with  sex and elderly age-bands using five year suicide data

**DOI:** 10.5249/jivr.v3i2.64

**Published:** 2011-07

**Authors:** Ajit Shah

**Affiliations:** ^a^University of Central Lancashire, Preston, United Kingdom and Consultant Psychiatrist, West London Mental Health NHS Trust, London, United Kingdom.

## Abstract

**Background:**

Suicide rates generally increase with age. A recent cross-national study, using one-year cross-sectional data on suicide rates identified regional and cross-national patterns for elderly suicide rates. However, findings from studies using one-year cross-sectional data on suicide rates may be open to bias because of random year on year fluctuations in elderly suicide rates.

**Methods:**

One-year average of suicide rates for both sexes in the age-bands 65-74 and 75+ years were calculated from data on suicide rates for five consecutive years ascertained from the World Health Organization. Cross-national variations were examined by segregating different countries into four quartiles of suicide rates.

**Results:**

There was wide cross-national variation in elderly suicide rates. Elderly suicide rates were the lowest in Caribbean and Arabic/Islamic countries, and the highest in central and eastern European, countries emerging from the former Soviet Union, some oriental and some west European countries.

**Conclusions:**

The regional and cross-national patterns for elderly suicide rates observed in this study were almost identical to a similar earlier study using one-year cross-sectional data on suicide rates. This suggests that the findings of both studies were accurate and robust, and potential explanations for the observed cross-national variations in elderly suicide rates requires further study.

## Introduction

In developing and developed countries the proportion of the elderly in the general population is increasing.^1^Suicide rates generally increase with age.^2,3^Suicides are emotive, distressing to family and professionals, lead to loss of economic productivity (although less likely in older people) and can lead to litigation. Thus, suicides in the elderly are an important public health concern. Comprehensive understanding of the substantial worldwide variation in population patterns of suicide may be critical in the understanding of potential distil risk and protective factors and for developing prevention programmes.^4^

Cross-national comparisons of  elderly  suicide  rates  can allow generation of potentially testable hypotheses about etiological factors.^5^Cross-national elderly suicide rates have been compared.^2,5-8^Elderly suicide rates for 1992  were low in Malta, Greece, Albania, Armenia, Tajikistan, Uzbekistan, Mexico and Columbia and high in Austria, Belarus, Denmark, Estonia, Finland, France, Hungary, Kazakhstan, Latvia, Lithuania, Russia, Slovenia, Switzerland and rural China.^2^Another recent cross-national study, using only one-year cross-sectional data on suicide rates, reported that there is wide cross-national variation in elderly suicide rates and elderly suicide rates were the lowest in Caribbean, central American and Arabic countries, and the highest in central and eastern European, some oriental and some west European countries.^5^However, the findings of the latter study, using only one-year cross-sectional data on suicide rates, may have erroneous because suicide rates can randomly fluctuate year on year.^9^

Therefore, by using a one-year average of data on suicide rates for five consecutive years, the following facets were examined in a cross-national study using data more recent than those used in the earlier study:^5^(i) comparison of elderly suicide rates across different countries; (ii) the comparison of suicide rates between elderly males and females; and, (iii) the comparison of suicide rates between  the “old-old” (75+ years) and the “young-old” (65-74 years) age-bands.

## Methods

**1.Data collection**

Data on suicide rates for males and females in the age-bands 65-74 and 75+ years was ascertained manually by the author for ICD 9 code of E54 and ICD-10 code of X60-X84 from the WHO website (http://www.who.int/whosis/ database/mort/table1.cfm). For a small number of countries only the raw figures for the number of suicides were available from the WHO website. Suicide rates for these countries were calculated by dividing the number of reported suicides by the population size in the relevant age-band and sex group available on the same website. Data were ascertained for the latest five consecutive years. The one-year average suicide rate was calculated by dividing the sum of suicide rates for the latest five consecutive years by five. The median (range) for the latest year for the suicide rate data was 2005 (1983-2007); the total number of countries with this data was 97.

**2.Data analysis**

Cross-national comparisons of elderly suicide rates were examined descriptively by segregating all the countries into the four quartiles of elderly suicide rates to observe any regional trends; these analysis were conducted for males and females in both the age-bands. Wilcoxon’s matched-pair signed-rank test was used to compare suicide rates between elderly males and females and between the age-bands 65-74 years and 75+ years across the different countries. SPSS version 16 was used for data nalysis.

## Results

Data on elderly suicide rates were available for 97 countries from the WHO website (names of these countries are listed in Tables 2-5). The median (range) suicide rate (per 100,000 population in the relevant group) for males aged 65-74 years, males aged 75+ years, females aged 65-74 years and females aged 75+ years was 19.6 (0-93.98), 29.3 (0-161.42), 4.6 (0-32.54) and 5.06 (0-71.36) respectively.

**1.Comparison of elderly suicide rates across different countries**

Table 1illustrates the cut-off suicide rates for each of  the four quartiles for males and females and for age-bands 65-74 years and 75+ years. Tables 2-5 illustrate the different countries in the four quartiles for males and females by age-bands 65-74 years and 75+ years.

**Table:** Table 1:**Cut-off suicide rates (per 100,000 population for the relevant group) for the four quartiles**

Quartile	Males 65-74	Males 75+	Females 65-74	Females 75+
First	<7.6	<8.9	<1.0	<0.92
Second	>7.6-19.6	>8.9-29.3	>1.0-4.6	>0.92-5.1
Third	>19.6-33.6	>29.3-56.2	>4.6-10.2	>5.1-15.0
Fourth	>33.6	>56.2	>10.2	>15.0

Suicide rates in males aged 65-74 years were the lowest in Caribbean (Anguilla, Antigua, Aruba, Bahamas, Bermuda, British Virgin Islands, Dominica, Jamaica, Montserrat, St Vincent and Grenadines Islands, and Turks and Caicos Islands), central American (Belize, Ecuador and Guatemala) and Arabic and Islamic countries (Bahrain, Kuwait and Brunei) countries. Suicide rates in males aged 65-74 years were the highest in central and east European and countries emerging from the former Soviet Union (Belarus, Bulgaria, Croatia, Estonia, Georgia, Hungary, Kazakhstan, Latvia, Lithuania, Moldavia, Russia, Slovenia and Ukraine), some oriental (South Korea and Japan), and  some west European (Austria, Belgium, France, Finland, Luxembourg and Switzerland) countries(Table 2).A similar pattern, with some variations, was also observed for suicide rates in males aged 75+ years, females aged 65-74 years and females aged 75+ years (Table 3,4,5).

**Table:** Table 2:**Suicide rates in males aged 65-74 years by quartiles and country (N=97)**

First	Second	Third	Fourth
Albania	Belize	Argentina	Austria
Anguilla	Brazil	Australia	Belarus
Antigua	Canada	Bosnia	Belgium
Armenia	Ecuador	Chile	Bulgaria
Aruba	El Salvador	Hong Kong	Costa Rica
Azerbaijan	French Guiana	Czech Republic	Croatia
Bahamas	Georgia	Denmark	Cuba
Bahrain	Iceland	Germany	Estonia
British Virgin Islands	Ireland	Kyrgyzstan	Finland
Brunei	Israel	Malta	France
Dominica	Italy	Norway	Georgia
Falkland	Mauritius	Poland	Hungary
Guatemala	Mexico	Portugal	Japan
Jamaica	Netherlands	Reunion	Kazakhstan
Kiribati	New Zealand	Romania	Latvia
Kuwait	Nicaragua	St Lucia	Lithuania
Maldives	Panama	Seychelles	Luxembourg
Montserrat	Paraguay	Singapore	Martinique
Peru	Saint Kitts & Nevis	Slovakia	South Korea
St Vincent & Grenadines	Surinam	Spain	Moldavia
South Africa	Turkmenistan	Sweden	Russia
Tajikistan	United Kingdom	Macedonia	Slovenia
Turks & Caicos	Venezuela	Trinidad	Switzerland
Bermuda	Greece	USA	Ukraine
		Guadeloupe	

**Table:** Table 3:**Suicide rates in males aged 75+ years by quartiles and country (N=97)**

First	Second	Third	Fourth
Albania	Australia	Argentina	Austria
Anguilla	Belize	Bosnia	Belarus
Antigua	Brazil	Brunei	Belgium
Armenia	Canada	Chile	Bulgaria
Aruba	Ecuador	Denmark	Costa Rica
Azerbaijan	El Salvador	French Guiana	Croatia
Bahamas	Georgia	Finland	Cuba
Bahrain	Greece	Guyana	Czech Rep
Bermuda	Guadeloupe	Italy	Estonia
British Virgin Islands	Ireland	Japan	France
Dominica	Israel	Macedonia	Germany
Falklands	Kyrgyzstan	Martinique	Hong Kong
Guatemala	Malta	Moldavia	Hungary
Iceland	Mauritius	Poland	Kazakhstan
Jamaica	Mexico	Portugal	South Korea
Kiribati	Netherlands	Reunion	Latvia
Kuwait	New Zealand	Romania	Lithuania
Maldives	Norway	Singapore	Luxembourg
Montserrat	Panama	Slovakia	Russia
Peru	Paraguay	Spain	Seychelles
Saint Kitts & Nevis	Turkmenistan	St Lucia	Slovenia
South Africa	United Kingdom	Surinam	St Vincent
Tajikistan	Venezuela	Sweden	Switzerland
Turks & Caicos	Nicaragua	Trinidad	Ukraine
		USA	

**Table:** Table 4:**Suicide rates in females aged 65-74 years by quartiles and country (N=97)**

First	Second	Third	Fourth
Anguilla	Albania	Argentina	Austria
Antigua	Armenia	Austria	Belarus
Aruba	Azerbaijan	Bosnia	Belgium
Bahrain	Bahamas	Canada	Bulgaria
Belize	Brazil	Czech Republic	Costa Rica
Bermuda	Chile	Denmark	Croatia
British Virgin Islands	Ecuador	Germany	Cuba
Brunei	Georgia	Guyana	Estonia
Dominica	Greece	Israel	Finland
El Salvador	Guadeloupe	Italy	France
Falklands	Ireland	Malta	Hong Kong
French Guiana	Iceland	Mauritius	Hungary
Guatemala	Kyrgyzstan	Netherlands	Japan
Jamaica	Martinique	Norway	Kazakhstan
Kiribati	Mexico	Poland	South Korea
Kuwait	New Zealand	Portugal	Latvia
Maldives	Nicaragua	Reunion	Lithuania
Montserrat	Panama	Romania	Luxembourg
Peru	Paraguay	Slovakia	Macedonia
Seychelles	Tajikistan	Slovenia	Moldavia
St Kitts & Nevis	United Kingdom	South Africa	Russia
St Lucia	USA	Spain	Singapore
St Vincent's & Grenadines	Venezuela	Surinam	Switzerland
Turks & Caicos		Sweden	Ukraine
		Trinidad	
		Turkmenistan	

**Table:** Table 5:**Suicide rates in females aged 75+ years by quartiles and country (N=97)**

First	Second	Third	Fourth
Anguilla	Australia	Albania	Austria
Antigua	Azerbaijan	Argentina	Belarus
Aruba	Brazil	Armenia	Belgium
Bahamas	Canada	Bosnia	Bulgaria
Bahrain	Chile	Costa Rica	Croatia
Belize	Ecuador	Finland	Cuba
Bermuda	El Salvador	Georgia	Czech Rep
British Virgin Islands	Greece	Guadeloupe	Denmark
Brunei	Guyana	Israel	Estonia
Dominica	Iceland	Italy	France
Falklands	Ireland	Kyrgyzstan	Germany
French Guiana	Martinique	Luxembourg	Hong Kong
Guatemala	Mauritius	Malta	Hungary
Jamaica	Mexico	Moldavia	Japan
Kiribati	New Zealand	Netherlands	Kazakhstan
Kuwait	Nicaragua	Poland	South Korea
Montserrat	Norway	Portugal	Latvia
Peru	Panama	Romania	Lithuania
Seychelles	Paraguay	Slovakia	Macedonia
Slovenia	Reunion	South Africa	Maldives
St Kitts & Nevis	Tajikistan	Spain	Russia
St Vincent's & Grenadines	United Kingdom	St Lucia	Singapore
Suriname	USA	Sweden	Switzerland
Turks & Caicos	Venezuela	Trinidad	Ukraine
		Turkmenistan	

**2. Comparison of suicide rates in males and females across countries**

Using the Wilcoxon’s matched-pair signed-rank test, suicide rates across different countries were significantly higher in males than females for the age-bands 65-74 years (Z=-7.79, P <0.00001) and 75+ years (Z=-7.69, P <0.00001).

**3. Comparison of suicide rates between the two age-bands**

Using the Wilcoxon's matched-pair signed-rank test, suicide rates across different countries were significantly higher in the age-band 75+ years than in the age-band 65-74 years in males (Z=-5.95, P < 0.00001) and females (Z = -4.88, P < 0.00001).

## Discussion

Some methodological issues need consideration. Data on suicide rates in cross-national studies should be viewed cautiously because: data were not available from all countries;^10,11^the validi of the data was unclear;^6,11^the legal criteria for the proof of suicide vary between countries and different regions within a country;^7,11^some countries have poor death registration facilities;^7^and, cultural and religious factors and stigma attached to suicide, particularly in some Arabic and Asian countries, may lead to under-reporting of suicides.^11,12^Additionally, some suicides may be misclassified as deaths due to undetermined causes,^13^but data on undetermined deaths were not available for all countries. Some of these methodological difficulties were reduced by using a one-year average of five consecutive year data on elderly suicide rates. Moreover, the latest and best available data set from the WHO were used.

The regional and cross-national patterns in elderly suicide rates observed in the current study were similar to those reported previously.^2,5^In particular, the observed regional and cross-national patterns in elderly suicide rates were almost identical to a recent study using similar methodology.^5^However, there were four important methodological differences between the current and the latter study. First, one-year average of five consecutive years data on elderly suicide were used in the current study, whereas one-year cross-sectional data on suicide rates were used in the previous study. This would serve to reduce any bias due to year on year random fluctuations in elderly suicide rates. Second, in the current study countries only reporting raw numbers of elderly suicides were also included and suicide rates were specifically calculated for those countries, whereas such countries were excluded in the previous study. Third, consequently, the sample size in the current study was significantly larger. Finally, the current study used much more recent data than the earlier study and thus also provides data on the current status of regional and cross-national patterns of elderly suicide rates. Moreover, the observations that suicide rates are higher in males than females for both the elderly age-bands and higher in the 75+ years age-band than 65-74 years age-band in males and females were also consistent with previous studies.^2,5^

The findings of the regional and cross-national patterns, the gender differences and age differences in elderly suicide rates in the current study and the previous study were almost identical despite the methodological improvements in the current study, and this suggests that the findings of both studies were accurate and robust. Therefore, the observed and persistent regional and cross-national patterns in elderly suicide rates requires further consideration because there may be several potential explanations including regional and cross-national variations in: genetic factors predisposing to mental illness and suicidal behaviors; the prevalence of mental illness (especially depression); life expectancy; socioeconomic deprivation and income inequality; social fragmentation; cultural and religious factors; availability of appropriate mental healthcare services; and presence of public health policy initiatives to reduce suicide rates.^3,5,14^Some studies addressing these issues have emerged, including socio-economic status, education, population growth, service provision and funding, elderly dependency ratios, household size and ageing,^3,14-21^
